# Influence of buccal and palatal bone thickness on post-surgical marginal bone changes around implants placed in posterior maxilla: a multi-centre prospective study

**DOI:** 10.1186/s12903-023-02991-3

**Published:** 2023-05-22

**Authors:** Marco Cicciù, Umberto Pratella, Luca Fiorillo, Fabio Bernardello, Francesco Perillo, Antonio Rapani, Claudio Stacchi, Teresa Lombardi

**Affiliations:** 1 School of Dentistry, Department of General Surgery and Surgical-Medical Specialties, University of Catania, Catania, 95124 Italy; 2 Private Practice, Bologna, 40138 Italy; 3 Multidisciplinary Department of Medical-Surgical and Odontostomatological Specialties, University of Campania “Luigi Vanvitelli”, Naples, 80121 Italy; 4 School of Dentistry, Aldent University, Tirana, 1001 Albania; 5 Private Practice, Terranegra di Legnago (VR), Legnago, 37045 Italy; 6 Private Practice, Naples, 80121 Italy; 7 Department of Medical, Surgical and Health Sciences, University of Trieste, Trieste, 34125 Italy; 8 Department of Health Sciences, Magna Graecia University, Catanzaro, 88100 Italy

**Keywords:** Dental implant, Bone remodeling, Bone tissue, Maxillary bone, Piezosurgery, Socket preservation

## Abstract

**Background:**

Numerous clinical variables may influence early marginal bone loss (EMBL), including surgical, prosthetic and host-related factors. Among them, bone crest width plays a crucial role: an adequate peri-implant bone envelope has a protective effect against the influence of the aforementioned factors on marginal bone stability. The aim of the present study was to investigate the influence of buccal and palatal bone thickness at the time of implant placement on EMBL during the submerged healing period.

**Methods:**

Patients presenting a single edentulism in the upper premolar area and requiring implant-supported rehabilitation were enrolled following inclusion and exclusion criteria. Internal connection implants (Twinfit, Dentaurum, Ispringen, Germany) were inserted after piezoelectric implant site preparation. Mid-facial and mid-palatal thickness and height of the peri-implant bone were measured immediately after implant placement (T0) with a periodontal probe and recorded to the nearest 0.5 mm. After 3 months of submerged healing (T1), implants were uncovered and measurements were repeated with the same protocol. Kruskal-Wallis test for independent samples was used to compare bone changes from T0 to T1. Multivariate linear regression models were built to assess the influence of different variables on buccal and palatal EMBL.

**Results:**

Ninety patients (50 females, 40 males, mean age 42.9 ± 15.1 years), treated with the insertion of 90 implants in maxillary premolar area, were included in the final analysis. Mean buccal and palatal bone thickness at T0 were 2.42 ± 0.64 mm and 1.31 ± 0.38 mm, respectively. Mean buccal and palatal bone thickness at T1 were 1.92 ± 0.71 mm and 0.87 ± 0.49 mm, respectively. Changes in both buccal and palatal thickness from T0 to T1 resulted statistically significant (p = 0.000). Changes in vertical bone levels from T0 to T1 resulted not significant both on buccal (mean vertical resorption 0.04 ± 0.14 mm; p = 0.479) and palatal side (mean vertical resorption 0.03 ± 0.11 mm; p = 0.737). Multivariate linear regression analysis showed a significant negative correlation between vertical bone resorption and bone thickness at T0 on both buccal and palatal side.

**Conclusion:**

The present findings suggest that a bone envelope > 2 mm on the buccal side and > 1 mm on the palatal side may effectively prevent peri-implant vertical bone resorption following surgical trauma.

**Trial Registration:**

The present study was retrospectively recorded in a public register of clinical trials (www.clinicaltrials.gov - NCT05632172) on 30/11/2022.

## Background

Marginal bone stability has always been considered as one of the main requisites for long-term success of osseointegrated dental implants [1]. In particular, early marginal bone loss (EMBL), a non-infective phenomenon occurring within the first year of implant function, seems to have a strong predictive value for future implant health. EMBL may result in treated implant surface exposure to the oral environment with micro-rough titanium surface facilitating bacterial biofilm adhesion and colonization. Galindo-Moreno and co-workers demonstrated that marginal bone loss ≥ 0.44 mm at 6 months post-loading is an indicator of peri-implant bone loss progression [2]. Windael and colleagues, in a 10-year prospective study conducted on 1482 implants, showed that EMBL ≥ 0.5 mm during the first year of function is associated to a 5.43 times higher odds for future peri-implantitis development [3]. Recently, EMBL < 0.5 mm within 6 months after prosthetic loading has been proposed as a distinctive and objective criterion of success in implantology [4].

Numerous clinical variables may influence EMBL, including implant design [5, 6], surgical trauma [7, 8], supracrestal tissue height establishment [9, 10], abutment height [11–13], multiple abutment disconnections [14, 15], presence of cement remnants [16, 17] and emergence profile angle of the prosthetic restoration [18, 19]. However, edentulous ridge width at implant placement site plays a crucial role: an adequate peri-implant bone envelope has a protective effect against the detrimental influence of the aforementioned factors on peri-implant marginal bone stability. In case of insufficient bone width, the clinician may perform horizontal ridge augmentation procedures [20, 21], use implants with reduced diameter [22, 23] or plan a combination of the above. Nevertheless, there is no agreement in the literature on the minimum amount of bone that should surround an implant. Belser et al. recommended a minimal thickness of 1 mm of the buccal bone after implant placement to prevent EMBL [24], Spray and co-workers suggested a minimal thickness of 1.8 mm [25], while other authors indicated safety thresholds of 2.0 mm [26–28] or even greater [29]. However, these recommendations come mainly from narrative reviews, expert opinions or from studies with no accurate control of the possible confounding factors. Different conclusions were drawn in a recent prospective study by Mehreb and co-workers, who demonstrated a relative dimensional stability even for initially thin (< 1 mm) buccal plates [30].

This substantial uncertainty is confirmed by two systematic reviews on this topic concluding that, despite the aforementioned recommendations, there is insufficient evidence to set a threshold for minimal buccal bone thickness necessary to ensure peri-implant marginal bone stability and optimal aesthetic outcomes [31, 32].

Therefore, the aim of the present multi-centre prospective study was to investigate the influence of buccal and palatal bone thickness at the time of implant placement on peri-implant bone remodeling during the submerged healing period, with a strict control of possible confounding factors.

## Methods

### Study protocol

This multi-centre prospective clinical study has been reported following STROBE (Strengthening the Reporting of Observational Studies in Epidemiology) guidelines. All the procedures were conducted following the principles outlined in the Declaration of Helsinki as revised in Fortaleza (2013) for investigations with human subjects. The study protocol was submitted and approved by the relevant Ethical Committee (Regione Calabria, Comitato Etico Sezione Area Centro, No. 200/2021), and retrospectively recorded in a public register of clinical trials (www.clinicaltrials.gov-NCT05632172) on 30/11/2022.

Patients included in the study where thoroughly informed about the protocol, including surgical procedures, follow-up visits and therapeutic alternatives. Patients signed an informed consent form and authorized the use of their personal data for research purposes.

### Selection criteria

Any patient presenting a single edentulism in the upper premolar area and requiring implant-supported rehabilitation was eligible for this study. Furthermore, patients had to comply with the following inclusion and exclusion criteria.

Inclusion criteria:

- age > 18 years;
- no smokers;
- no history of periodontal disease;
- healed bone crest (at least six months elapsed from tooth extraction);
- crestal bone height ≥ 8 mm;
- crestal bone with a minimum bucco-palatal width of 6 mm with no concomitant or previous bone augmentation procedures.

Exclusion criteria:

- absolute contraindications to implant surgery [33];
- immunological or genetic disorders;
- uncontrolled diabetes (HBA1c > 7.5%);
- present or past treatment with anti-resorptives;
- oncologic patients;
- history of head and/or neck radiotherapy;
- alcohol or drugs abuse;
- implant insertion torque > 50 Ncm.

All patients were recruited and treated between September 2020 and November 2021 in four clinical centres by four independent operators (M.C.; U.P.; F.B.; F.P.).

### Surgical Procedure

**Fig. 1:**
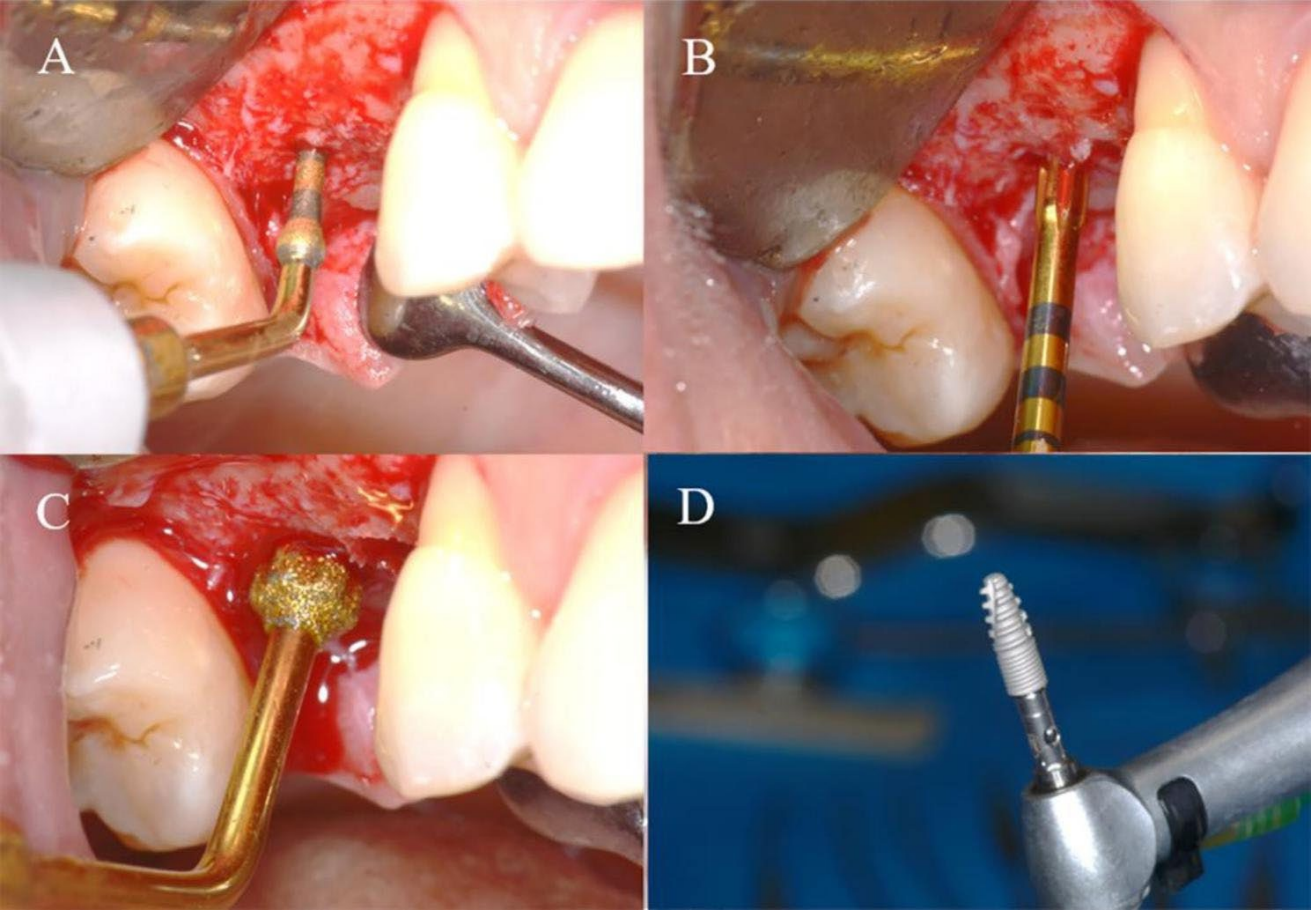
Implant site preparation with ultrasonic inserts. (A) Pilot osteotomy with IM1 insert; (B) Implant osteotomy was enlarged at 2 mm diameter with IM2 insert; (C) Final preparation of the cortical bone with IP 3-4 insert; (D) The implant used in the present study (Twinfit, Dentaurum, Ispringen, Germany)

**Fig. 2:**
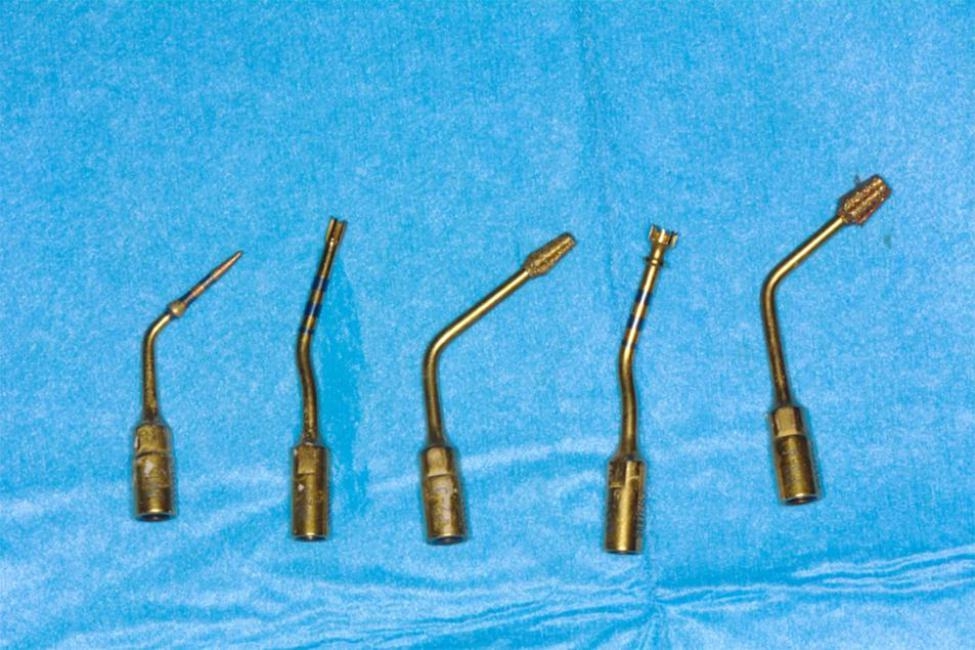
The sequence of piezoelectric inserts used for implant site preparation. Left to right: IM1, IM2, IP2-3; IM3; IP3-4 (Mectron, Carasco, Italy)

All patients received antibiotic prophylaxis (amoxicillin 2 g one hour before surgery). Under local anesthesia (articaine 4% with epinephrine 1:100.000), a full-thickness envelope flap was elevated and bucco-palatal crestal bone width was measured with a Iwanson caliper (KLS Martin Group, Umkirch, Germany) 1 mm below the crestal level at programmed implant site. Implant osteotomy was then prepared with specific piezoelectric tips (Piezosurgery Touch, Mectron, Carasco, Italy) under abundant irrigation of cold saline solution (Fig. 1). Tip sequence was the following: IM1, IM2, IP2-3, IM3, IP3-4 (Fig. 2). Then, a 3.7 mm diameter implant with internal connection was inserted (Twinfit, Dentaurum, Ispringen, Germany) and insertion torque was recorded by the surgical motor (Implantmed, W&H, Burmoos, Austria).

Following implant insertion, mid-facial and mid-palatal thickness of the peri-implant bone were measured at crestal level with a periodontal probe (UNC 15, Hu-Friedy, Chicago, USA) and recorded to the nearest 0.5 mm. Additionally, the distance between crestal bone level and implant platform was measured with the same probe to the nearest 0.5 mm at mid-facial and mid-palatal aspect of each implant. These values were considered negative when implant platform was placed under bone level.

Cover screw was then positioned and flaps were sutured with synthetic monofilament to attain first intention closure and allow submerged healing of the implant. Antibiotic therapy (amoxicillin 1 g / 2 times a day) was prescribed for six days and sutures were removed 10 days after surgery.

After three months of healing, second stage surgery was performed and crestal width and height were measured following the aforementioned procedure. Implants were then connected with healing abutments and flaps were sutured with synthetic monofilament. Implants were restored with screwed metal-ceramic crowns and followed for one year after prosthesis delivery.

### Statistical analysis

Data were analyzed by an independent investigator (A.R.) using a statistical software (STATA 16.0, StataCorp, College Station, USA), with the patient considered as the statistical unit (one implant per patient). Data were expressed as mean ± standard deviation and the significance level was set at α = 0.05.

The primary outcome of the present study was the vertical variation of peri-implant buccal and palatal bone level from implant placement (T0) to uncovering stage (3 months later – T1). Data normality was assessed using Shapiro-Wilk test. As data normality could not be assumed, Kruskal-Wallis test for independent samples was used to compare bone changes from T0 to T1. Multivariate linear regression models were built to assess the influence of different variables on buccal and palatal EMBL.

The present paper has been checked with the Fi-Index tool on November 21, 2022 according to Scopus.com and obtained a score of 0.25 for the first, last and corresponding author only [34, 35].

## Results

Ninety-two patients, fulfilling inclusion and exclusion criteria, were recruited in the present study and underwent implant placement. Two patients did not present for implant uncovering at the prescribed time-point (after three months of submerged healing) and dropped out from the study. A total of 90 patients (50 females, 40 males, mean age 42.9 ± 15.1 years, age range 19–65), treated with the insertion of 90 implants in maxillary premolar area, were included in the final analysis. No intra- or post-operative complications or implant failure were recorded at the follow-up scheduled after 12 months of function.

Mean crestal bone thickness at T0 was 7.43 ± 0.93 mm. After implant insertion, mean buccal and palatal bone thickness were 2.42 ± 0.64 mm and 1.31 ± 0.38 mm, respectively. Mid-buccal and mid-palatal bone height at T0 were − 0.39 ± 0.48 mm and − 0.59 ± 0.48 mm, respectively. Implants were inserted with a mean insertion torque of 34.7 ± 5.6 Ncm.

**Fig. 3:**
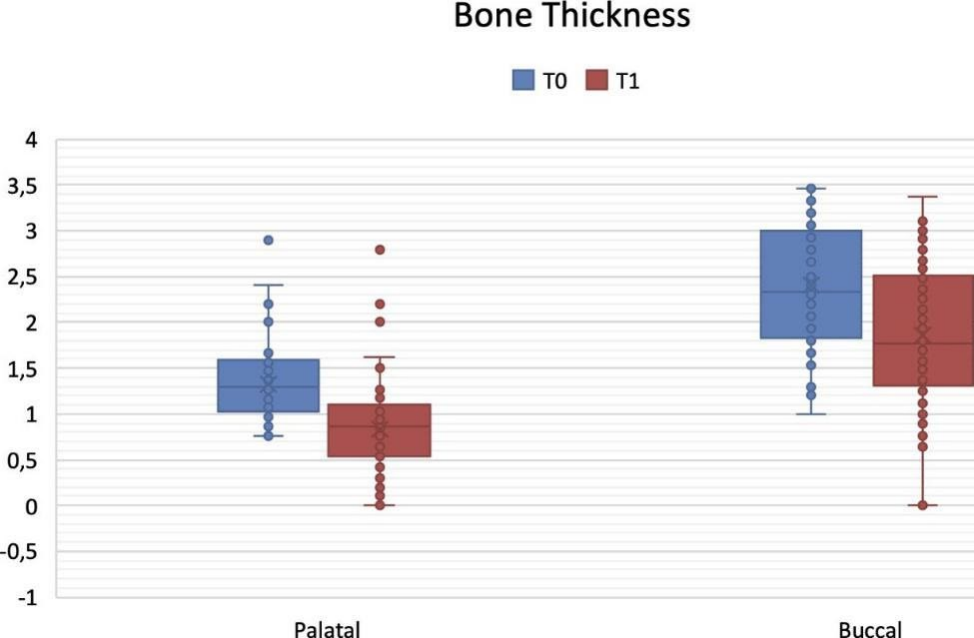
Variations in palatal and buccal bone thickness from T0 (implant insertion) to T1 (implant uncovering). Data are expressed in mm

Mean buccal and palatal bone thickness at T1 were 1.92 ± 0.71 mm and 0.87 ± 0.49 mm, respectively. Changes in both buccal and palatal thickness from T0 to T1 resulted statistically significant (p = 0.000) (Fig. 3).

**Fig. 4:**
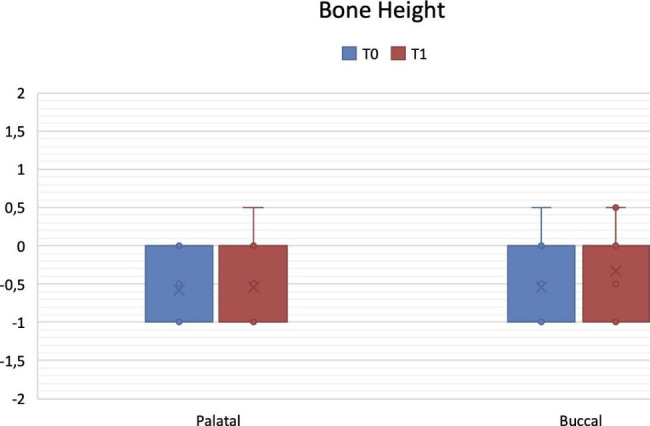
Variations in palatal and buccal bone height from T0 (implant insertion) to T1 (implant uncovering). Data are expressed in mm

Mid-buccal and mid-palatal bone height at T1 were − 0.34 ± 0.52 mm and − 0.56 ± 0.50 mm, respectively. Changes in vertical bone levels from T0 to T1 resulted not significant both on buccal (mean vertical resorption 0.04 ± 0.14 mm; p = 0.479) and palatal side (mean vertical resorption 0.03 ± 0.11 mm; p = 0.737) (Fig. 4)

Multivariate linear regression analysis showed a significant negative correlation between vertical bone resorption and bone thickness at T0 on both buccal and palatal side. Complete results are presented in Table 1.

**Table 1:** Multivariate linear regression models assessing the influence of different variables on vertical bone resorption

	Buccal bone height resorption		Palatal bone height resorption	
Mean ± SD	0.04 ± 0.14 mm		0.03 ± 0.11 mm	
**Variables**	**p-value**	**C.I. (95%)**	**p-value**	**C.I. (95%)**
Gender	0.061	0.112–0.003	0.889	-0.053–0.046
Age	0.390	0.003–0.001	0.773	-0.002–0.001
Insertion torque	0.253	0.002–0.008	0.207	-0.002–0.007
Implant length	0.656	0.035–0.022	0.919	-0.026–0.023
B Thickness T0	0.003*	0.025–0.113	NA	NA
P Thickness T0	NA	NA	0.037*	-0.129–0.004

SD: standard deviation; C.I.: confidence interval; B: buccal; T0: at implant insertion; P: palatal; NA: not applicable

## Discussion

The present study focused on dimensional changes of peri-implant buccal and palatal bone occurring during the submerged healing period, from implant placement to uncovering stage (3 months after insertion). In the present sample, which included only single implants placed in the upper premolar area, a significant horizontal reduction was recorded on both buccal and palatal side, together with a substantial stability of vertical bone levels.

Buccal and palatal bone thickness decreased of about 0.4–0.5 mm from T0 to T1, in perfect agreement with previous studies on the same topic [30, 36] and in partial agreement with Spray and co-workers (2000), who reported greater mean buccal bone loss (0.7 mm) from implant insertion to uncovering stage [25].

Muco-periosteal flap elevation and exposure of the underlying alveolar crest stimulates osteoclastic activity resulting in bone resorption. This phenomenon has been already observed in periodontal field: two clinical studies recorded a mean horizontal bone resorption of 0.5–0.6 mm after full-thickness elevation during apically positioned flap [37, 38]. Most recently, Fickl and co-workers demonstrated that, when elevating a full-thickness flap during extractive surgery, an additional 0.7 mm of horizontal bone resorption may be expected [39].

Cortical bone vascularization derives from the periosteal vessels (outer supply) and from the endosteum (inner supply) [40]. When performing an open-flap implant insertion, periosteal elevation jeopardizes the external blood supply to the cortical bone and the presence of the implant compromises the internal blood supply from the endosteum. This critical situation may be worsened by excessive surgical trauma such as overheating or compression necrosis (i.e., high insertion torque). In cases of narrow ridges, limited blood supply and absence of spongious bone may result in an insufficient osteoblastic cell density in the bone remodeling area, where bone resorption becomes the prevalent activity [41, 42].

In the present study, mean crestal bone width at implant placement was 7.43 ± 0.93 mm. A slightly palatal positioning of the implant was adopted to increase the amount of bone on the buccal side: mean thickness of buccal and palatal plates at implant insertion were 2.42 ± 0.64 mm and 1.31 ± 0.38 mm, respectively. Implant site preparation was performed using piezoelectric instruments, in order to exploit the favorable healing potential following ultrasonic bone surgery [43–45]. Moreover, an upper threshold of 50 Ncm was fixed for implant insertion torque, to avoid the detrimental effect of over-compression of the cortical bone [46, 47]. A recent study by Coyac et al. [8] showed that excessive compression forces produce extensive damage to the peri-implant bone matrix, triggering osteocyte apoptosis and creating a wider necrotic area around the implant. Furthermore, a high cortical/cancellous bone ratio (typical of narrow ridges) increases site susceptibility to extensive bone resorption.

Multivariate analysis showed that, among all the considered variables, only buccal and palatal bone thickness at baseline demonstrated a significant negative correlation with vertical bone resorption. In the present sample, substantial stability of buccal and palatal vertical bone levels during the submerged healing period was demonstrated (mean buccal vertical resorption 0.04 ± 0.14 mm; mean palatal vertical resorption 0.03 ± 0.11 mm). These results suggest that a bone envelope > 2 mm on the buccal side and > 1 mm on the palatal side may effectively prevent peri-implant vertical bone resorption following surgical trauma. This finding is in perfect agreement with a very recent systematic review, concluding that thin buccal bone at implant placement (< 2 mm) favors post-operative bone changes that may compromise the integrity of the buccal plate, leading to biologic and esthetic complications [48]. However, it must be considered that, after implant uncovering, other factors will affect peri-implant marginal bone stability during the first year of function (supracrestal tissue height establishment [9, 10], abutment height [11–13], multiple abutment disconnections [14, 15], presence of cement remnants [16, 17] and emergence profile angle of the restoration [18, 19]). The presence of adequate buccal and palatal bone thickness is a crucial point to maintain stability of vertical bone levels also during the action of the aforementioned factors.

Some limitations should be considered when interpreting the outcomes of the present study. The selection of surgical sites in a specific area (only upper premolars) and the use of a single implant type with specific features limit the generalization of the present results. Furthermore, the method used to assess marginal bone levels (periodontal probe) represents another limitation, as this approach has a limited sensitivity (0.5 mm) in detecting marginal bone variations. Even if this measurement method could influence the results considering the magnitude of the possible error (0.1–0.2 mm), it was adopted by the great majority of the previous studies conducted on this topic [25, 27, 28]. Merheb and co-workers [30] designed a device composed by a metallic structure guiding the measuring tool (a blunt needle) soldered to an abutment specific for the implant used in their study. Authors declared that this method could suffer from an intrinsic imprecision of 0.08–0.1 mm and that measurements may be flawed by intra-observer and inter-observer variability. Additionally, this method was applied only on the buccal side of the implant. Another option is the measurement of peri-implant buccal and palatal bone thickness on CBCT images. Even if, using adequate informatics tools, inter- and intra-observer reproducibility has been demonstrated to be reliable with this technique [49], background scattering and problems with standardization of the measurements are frequently encountered [50]. It should be also considered that, with this last measurement option, patient is subjected to additional radiation exposure, not necessary for therapeutic reasons.

## Data Availability

The dataset of the analyzed data is available from the corresponding author (Rapani Antonio) upon reasonable request.

## Abbreviations

EMBL: Early marginal bone loss
HBA1c: Glycated hemoglobin
